# Longitudinal Changes in Magnetic Resonance Spectroscopy in Pediatric Concussion: A Pilot Study

**DOI:** 10.3389/fneur.2019.00556

**Published:** 2019-06-07

**Authors:** Erin J. Meyer, Jeffrey N. Stout, Ai Wern Chung, P. Ellen Grant, Rebekah Mannix, Borjan Gagoski

**Affiliations:** ^1^Department of Medicine, Boston Children's Hospital, Harvard Medical School, Boston, MA, United States; ^2^Fetal Neonatal Neuroimaging and Developmental Science Center, Boston Children's Hospital, Boston, MA, United States; ^3^Division of Newborn Medicine, Department of Medicine, Boston Children's Hospital, Harvard Medical School, Boston, MA, United States; ^4^Department of Radiology, Boston Children's Hospital, Harvard Medical School, Boston, MA, United States; ^5^Division of Emergency Medicine, Boston Children's Hospital, Boston, MA, United States; ^6^Department of Pediatrics, Department of Emergency Medicine, Harvard Medical School, Boston, MA, United States

**Keywords:** pediatric, adolescent, mild traumatic brain injury, concussion, magnetic resonance spectroscopy

## Abstract

**Background:** Nearly 20% of US adolescents report at least one lifetime concussion. Pathophysiologic models suggest that traumatic biomechanical forces caused by rotational deceleration lead to shear stress, which triggers a neurometabolic cascade beginning with excitotoxicity and leading to significant energy demands and a period of metabolic crisis for the injured brain. Proton magnetic resonance spectroscopy (^1^H MRS) offers a means for non-invasive measurement of neurometabolic changes after concussion.

**Objective:** Describe longitudinal changes in metabolites measured *in vivo* in the brains of adolescent patients with concussion.

**Methods:** We prospectively recruited 9 patients ages 11 to 20 who presented to a pediatric Emergency Department within 24 h of concussion. Patients underwent MRI scanning within 72 h (acute, *n* = 8), 2 weeks (subacute, *n* = 7), and at approximately 1 year (chronic, *n* = 7). Healthy, age and sex-matched controls were recruited and scanned once (*n* = 9). ^1^H MRS was used to measure N-acetyl-aspartate, choline, creatine, glutamate + glutamine, and myo-inositol concentrations in six regions of interest: left and right frontal white matter, posterior white matter and thalamus.

**Results:** There was a significant increase in total thalamus glutamate+glutamine/choline at the subacute (*p* = 0.010) and chronic (*p* = 0.010) time points, and a significant decrease in total white matter myo-inositol/choline (*p* = 0.030) at the chronic time point as compared to controls.

**Conclusion:** There are no differences in ^1^H MRS measurements in the acute concussive period; however, changes in glutamate+glutamine and myo-inositol concentrations detectable by ^1^H MRS may develop beyond the acute period.

## Introduction

Nearly 20% of US adolescents report experiencing at least one lifetime concussion, also referred to as mild traumatic brain injury (TBI), and concussion accounts for approximately 200,000 visits annually to pediatric emergency rooms (1, 2). Current guidelines for assessing and diagnosing pediatric concussion rely on clinical judgment without the use of routine laboratory or imaging tests (3, 4). While most children recover within 1 month, 20–30% will experience persistent symptoms beyond 1 month (5, 6). It remains unknown why some patients experience prolonged symptoms while others do not. Investigations into the pathophysiologic underpinnings of concussion are crucial to guide development of tools that will allow researchers and clinicians to more accurately diagnose, prognose, and guide future interventions.

Animal models suggest concussion results from shear stress caused by rotational deceleration from traumatic biomechanical forces (7). This shear stress triggers a neurometabolic cascade that begins with excitotoxicity and leads to significant energy demands and a period of metabolic crisis for the injured brain (8, 9). Non-invasive neuroimaging-based biomarkers have provided important insights into structural and functional brain abnormalities in concussion. For example, studies using advanced neuroimaging techniques in adults have shown that concussion can be associated with alterations in white matter (WM) microstructure (diffusion tensor imaging, DTI), functional connectivity (fMRI), and cerebral blood flow (MR angiography) (10, 11).

Proton magnetic resonance spectroscopy (^1^H MRS) allows direct investigation of metabolic shifts that occur during the neurometabolic cascade of concussion. ^1^H MRS enables non-invasive measurement of metabolite and neurotransmitter concentrations in brain tissue. Metabolites with distinctive prominent spectra in ^1^H MRS are N-acetyl aspartate (NAA), choline-containing compounds (Cho), and creatine-containing compounds (Cr). NAA is an amino acid highly concentrated in neurons, often interpreted as a marker of neuronal integrity and density, though some have shown dynamic changes in neuronal concentrations, suggesting a role as an indirect marker of the neuronal energy state (12, 13). Cho is a marker for cellular membrane turnover. Cr is a marker for cellular energetic systems and has also been proposed to have trophic and neuroprotective roles (14, 15). ^1^H MRS can also be used to measure neurotransmitter concentrations (e.g., γ-aminobutyric acid [GABA], glutamate), lactate, and myo-inositol (Ins), among many other molecules with subtler, more difficult to resolve spectra. One meta-analysis of ^1^H MRS data in adult TBI show decreased NAA, NAA/Cr and increased Cho/Cr at the subacute and chronic time points in moderate to severe cases, but did not reveal significant differences in mild TBI (16). One notable study, excluded from this meta-analysis, demonstrated decreased NAA/Cr at the acute post-concussive time point that normalized by 1 month (17).

Fewer neuroimaging studies have focused on pediatric concussion (18). Systematic reviews of neuroimaging in pediatric concussion have shown that DTI, fMRI and angiography findings do not always align with changes detected in adults (18–20). The adolescent brain may be uniquely vulnerable to the neurometabolic cascade, and this population requires special attention. Only a few studies, to our knowledge, have used in ^1^H MRS in the adolescent population, and the methodology and reported results are highly variable (21–25).

The purpose of our study was to use magnetic resonance spectroscopy imaging (MRSI), an advanced type of ^1^H MRS that allows multi-voxel measurements as compared to single-voxel spectroscopy, to explore possible differences in an extensive array of brain metabolites in pediatric patients with concussion as compared to controls. We further explored longitudinal trends in metabolites within concussed patients. Given the variable findings in preceding studies, this study was exploratory in nature. Nonetheless, we hypothesized that findings would be relatively consistent with observations in adult concussion as described above.

## Methods

### Subjects

This study was approved by the Institutional Review Board at Boston Children's Hospital and written informed consent was obtained from parent proxies for all subjects. We prospectively recruited patients who presented to a pediatric Emergency Department within 24 h of concussion. Each patient underwent MRI scanning within 72 h (acute), 2 weeks (subacute), and approximately 1 year (chronic) after the concussion. Concussion was defined as a blunt, sports-related injury to the head resulting in either (1) alteration in mental status (including loss of consciousness, disorientation, or amnesia) or (2) any of the following symptoms that started within 4 h of injury and were not present before the injury: headache, nausea, vomiting, dizziness/balance problems, fatigue, drowsiness, blurred vision, memory difficulty or difficulty concentrating. Patients were excluded from the study if they presented to the Emergency Department with Glasgow Coma Scale < 14, focal symptoms or other indications for head imaging or intracranial hemorrhage seen when imaging was obtained, orthopedic fracture, co-existing intra-abdominal or intra-thoracic trauma, or spinal-cord injury, or an underlying neurologic disorder or psychiatric illness requiring medications. Healthy, age and sex-matched controls were recruited and scanned once. Controls were recruited without current neurological complaints or recent head trauma at least a year prior to scanning.

### MRI Image Acquisition

Structural T1-weighted and MRSI volumes were collected at each time point. Imaging data were acquired on a 3T Siemens Tim Trio system (Erlangen, Germany), equipped with 32-channel receive coil array and maximum gradient strength and slew rate of 40 mT/m and 180 mT/m/ms, respectively. T1-weighted motion mitigated multi-echo MPRAGE (26) parameters were: TR = 2520 ms; TE = 1.74, 3.54, 5.34, and 7.14 ms; inversion time = 1,350 ms; field-of-view (FOV) = 240 mm^2^; voxel size = 1 mm^3^. For MRSI, we used an accelerated 3D MRSI sequence with spiral k-space trajectories (27), which has been fully implemented on Siemens MR platforms (28). This sequence provides for the first time in clinical settings volumetric CSI coverage of isotropic voxel size of 2 cc in 1 min of acquisition time. Specific imaging parameters were: Matrix size (x, y, z, f) = (13, 13, 8, 512), zero-padded to (16, 16, 8, 512) and encoded over FOV_XY_ = 16 cm^2^, FOV_Z_ = 10 cm, FOV_f_ = 1.2 KHz for an overall isotropic voxel size of 2 cc; excitation box was prescribed entirely within the brain to avoid lipid contamination from the skull, such that the center of the excitation box is centered around basal ganglia; TE = 30 ms, TR = 1.8 s, number of averages = 8 (for signal-to-noise-ratio [SNR] purposes), for an overall acquisition time of 7:48 min. An additional spectroscopy scan with no water suppression and with one average and TR = 1 s (overall acquisition time of 36 s) was acquired for the purposes of obtaining absolute quantitative estimates of brain metabolites.

### MRI Image Processing

MRSI was used to measure NAA, Cho, Cr, glutamate + glutamine (Glx), Ins concentrations. MRSI data was analyzed using TARQUIN and custom Matlab (Natick, MA, USA) routines (29). We obtained a 3D MRSI acquisition with a single short TE (30 ms) and without any spectral editing, which meant that GABA and lactate signals were not analyzed/fitted due to their inherent strong coupling with other metabolites in the spectra. Spectra from individual voxels were fit using default TARQUIN parameters, except the chosen reference signals were NAA, Cr, Cho, and Lipids, and the excitation scheme was LASER(30). Absolute concentrations were calculated assuming a water attenuation factor of 0.7 (metabolite T_2_ = 200 ms, water T_2_ = 80 ms, TE = 30 ms) and water concentration of 35,880 mM (WM water concentration). Concentration ratios were also determined for each metabolite concentration relative to Cho. Data quality was evaluated on a voxel-wise basis by first visually inspecting a selection of spectra to determine SNR and Q thresholds so that low quality spectra are rejected from the remaining dataset. SNR was determined by TARQUIN by taking the ratio between the maximum in the spectrum minus baseline divided by two times the root mean squre of the residual between 0.5–4 ppm. Q is a measure of how well the spectrum was fit by TARQUIN determined by the ratio of the fit residual to the noise level of the spectrum, specifically the standard deviation of the fit residual (between around 0.5–4 ppm) divided by the standard deviation of the noise region.

**Figure 1:**
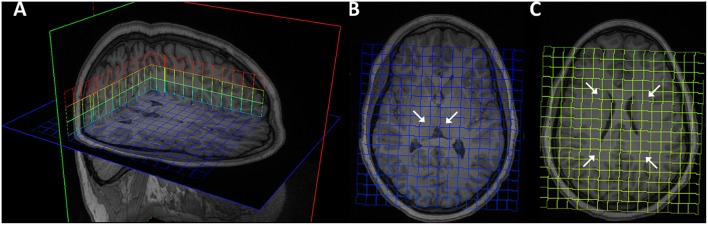
Magnetic resonance spectroscopy excitation grid overlaying T1 MPRAGE images of representative brain. **(A)** 3D visualization. **(B)** Bilateral thalamus ROIs indicated by arrows on transverse slice. **(C)** Bilateral frontal and parietal ROIs.

For each patient and time point, the 3D MRSI excitation grid was overlaid onto the T1-weighted structural volume to allow visualization and manual selection of region of interest (ROI). Six MRSI voxels were selected as each regions of interest: left and right frontal WM, posterior WM and thalamus (Figure 1). Given the large voxel size in MRSI, voxels were selected to include as much of the desired ROI as possible. For each subject, measurements from individual thalamus and WM voxels were averaged to create total thalamus and total WM meta-ROIs, respectively. T1-weighted images were pre-processed with the FreeSurfer “recon-all” pipeline (https://surfer.nmr.mgh.harvard.edu) and output were visually inspected, yielding four whole brain segmentation masks of the WM, cerebrospinal fluid (CSF), cortical gray matter (cGM), and subcortical gray matter (sGM) in native space. sGM comprised bilaterally the thalamus, caudate, putamen, pallidum, hippocampus, amygdala, and insula. For each MRSI ROI, the percentages of WM, CSF, cGM, and sGM within the ROI were computed.

### Statistical Analyses

All statistical analyses were performed using STATA statistical software package (StataCorp. 2017. Stata Statistical Software: Release 15. College Station, TX: StataCorp LLC). To exclude low-quality data, voxels were removed if SNR was <5 and the Q-statistic was >2.5. Fisher's exact test was used to analyze for sex differences between concussed and control patients. ANOVAs were used to analyze differences in age and partial volumes. Differences in metabolite concentrations at each time point were compared between patients and controls using Mann-Whitney U-tests. We first looked for differences in the meta-ROIs, and if a trend (*p* < 0.1) was detected, we further investigated for differences in the individual ROIs and for longitudinal trends. To assess longitudinal trends within the concussed patients, we used linear mixed effects models fit by maximum likelihood with metabolite concentration as the outcome and fixed and random effects of time point. Separate models were performed for each metabolite and ROI combination of interest. A threshold of *p* <0.05 was used to determine statistical significance. Due to the exploratory nature of our pilot study, *p*-values were not corrected for multiple comparisons.

## Results

### Demographics

Baseline group demographics and ROI partial volume data are presented in Table 1. In total, spectroscopy data was collected on nine concussed subjects and nine controls. Due to inability to collect MRSI data on every subject, it must be noted that these cohorts were not perfectly age-matched. After exclusion of corrupted data and accounting for loss to follow up, six concussion subjects had full longitudinal data at all three time points, one had data at the acute and chronic time points, and two subjects had only a single time point (one acute, and one subacute). Among all voxels from all participants, only five were excluded from analysis due to poor quality spectra (SNR < 5 and Q > 2.5). There were no significant differences in sex or age between concussion and control groups. There were no significant partial volumes differences between groups.

**Table 1:** Demographics and ROI partial volumes.

	**Control (*n* = 9)**	**Concussion (*n* = 9)**	***p*-value**
Age, mean years (SD)	13.52 (1.26)	14.23 (2.81)	0.50
Female sex, *n* (%)	1 (11%)	1 (11%)	> 0.99
L thalamus, % sGM, mean (SD)	80.43 (14.90)	80.43 (12.14)	> 0.99
R thalamus, % sGM, mean (SD)	82.97 (17.19)	78.22 (11.23)	0.52
L frontal WM, % WM, mean (SD)	93.44 (6.17)	91.70 (10.97)	0.69
R frontal WM, % WM, mean (SD)	98.25 (1.97)	98.11 (1.60)	0.88
L parietal WM, % WM, mean (SD)	96.72 (2.54)	91.42 (10.73)	0.17
R parietal WM, % WM, mean (SD)	95.93 (3.01)	93.81 (5.50)	0.33

*Partial volumes refer to the first (baseline) collection time point. ROI, region of interest; SD, standard deviation; L, left; R, right; sGM, subcortical gray matter; WM, white matter*.

#### MRSI Analyses

**Figure 2:**
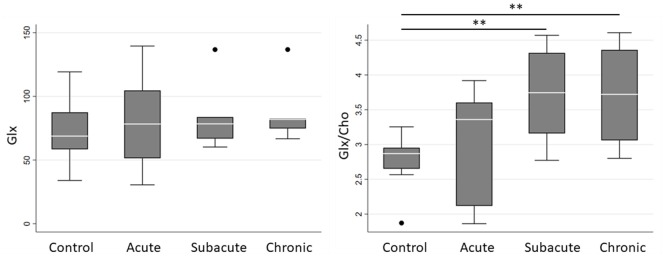
Boxplot representations comparing median and interquartile range for total thalamus Glx and Glx/Cho between controls and concussed patients at acute, subacute and chronic time points. Individual horizontal bars are presented for each significant group comparison. Glx = glutamate + glutamine, Cho = choline compounds. **P < 0.05 by Mann-Whitney U-test.

**Figure 3:**
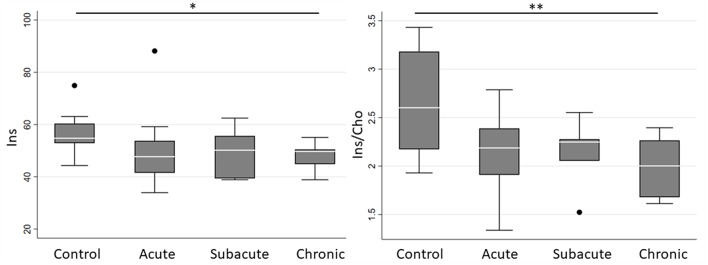
Boxplot representations comparing median and interquartile range for total white matter Ins and Ins/Cho between controls and concussed patients at acute, subacute and chronic time points. Individual horizontal bars are presented for each significant group comparison. Ins = myo-inositol, Cho = choline compounds. *P < 0.1, **p < 0.05 by Mann-Whitney U-test.

Spectroscopic measurements are presented in Table 2. On examination of the meta-ROIs, there was a significant increase in total thalamus Glx/Cho at the subacute (*p* = 0.010) and chronic (*p* = 0.010) time points as compared to controls (Figure 2). For total WM, there was a significant decrease in Ins/Cho (*p* = 0.030) and a trending decrease in Ins (*p* = 0.050) at the chronic time point (Figure 3).

**Table 2:** Median metabolite concentrations (mM), or concentrations ratios, and interquartile range by group and time point.

		**Control**	**Concussion**			**CRLB**
			**Acute (*n* = 8)**	**Subacute (*n* = 7)**	**Chronic (*n* = 7)**	
Days since injury, mean (SD)		7.78 (4.29)	1.13 (0.64)	16.14 (2.73)	417.86 (19.14)	
Cho	Total WM	22.21 (2.87)	22.43 (3.80)	24.04 (5.68)	24.72 (4.60)	6.67%
	Total thalamus	25.19 (6.54)	26.01 (13.52)	21.66 (8.74)	21.80 (11.38)	
Cr	Total WM	71.87 (11.15)	61.88 (40.68)	69.69 (10.20)	66.31 (6.16)	7.07%
	Total thalamus	71.20 (17.80)	69.49 (11.56)	61.72 (11.49)	63.37 (15.47)	
NAA	Total WM	104.26 (16.22)	101.12 (9.22)	109.33 (8.11)	102.96 (4.02)	6.72%
	Total thalamus	92.54 (19.74)	90.28 (34.58)	96.50 (42.43)	82.55 (27.36)	
NAA/Cho	Total WM	4.87 (0.92)	4.72 (0.47)	4.86 (0.84)	4.43 (0.69)	–
	Total thalamus	3.30 (1.11)	3.53 (0.90)	3.50 (1.44)	3.79 (1.45)	
Glx	Total WM	72.75 (9.23)	65.07 (31.19)	63.87 (21.36)	70.34 (12.84)	16.20%
	Total thalamus	68.80 (29.00)	78.29 (53.18)	78.40 (16.82)	81.89 (7.73)	
Glx/Cho	Total WM	3.57 (1.01)	2.83 (0.55)	2.98 (0.49)	2.87 (0.70)	–
	Total thalamus	2.87 (0.30)	3.36 (1.49)	^**^3.75 (1.16)	^**^3.72 (1.30)	
Ins	Total WM	54.75 (7.50)	47.71 (12.26)	50.17 (16.25)	^*^49.71 (5.64)	12.91%
	Total thalamus	44.15 (16.90)	41.45 (22.16)	48.37 (20.92)	42.51 (11.62)	
Ins/Cho	Total WM	2.35 (0.89)	2.28 (1.06)	2.33 (0.69)	^**^1.87 (0.35)	–
	Total thalamus	1.82 (0.29)	1.59 (0.41)	2.06 (0.45)	1.88 (1.20)	

*Total WM is the average of the 4 individual WM ROIs and total thalamus is the average of the 2 individual thalamic ROIs. SD, standard deviation; WM, white matter; Cho, choline compounds; Cr, creatine compounds; NAA, N-acetyl-aspartate; Glx, glutamate + glutamine; Ins, myo-inositol; CRLB, Cramér-Rao-lower-bound*.

* P < 0.1,

** *p < 0.05 by Mann-Whitney U-test*.

Further targeted investigation into the individual ROIs revealed a significant increase in left thalamus Glx/Cho at subacute (*p* = 0.017) and chronic (*p* = 0.039) time points. There was a significant decrease in right frontal WM Ins/Cho (*p* = 0.011) at the chronic time point, and a trending decrease in left frontal WM Ins/Cho (*p* = 0.064) at the chronic time point. There were no other significant differences in metabolite absolute concentrations or ratios for any of the individual or meta-ROIs at any time point when comparing concussed patients to controls.

Within the concussed groups, there was a significant linear increase over time in total thalamus Glx/Cho (*b* = 0.37, CI: 0.03–0.71, *p* = 0.034) and in total thalamus Ins/Cho (*b* = 0.46, CI: 0.11–0.81, *p* = 0.010). There were no significant linear trends over time in total WM Glx, Glx/Cho, Ins, or Ins/Cho.

## Discussion

This study used MRSI to broadly explore longitudinal changes in the neurometabolic state of the brain after pediatric concussion. Our study demonstrates the feasibility of using ^1^H MRS in the pediatric population to measure a range of neurometabolites. Our analyses suggest that Glx/Cho in the thalamus may be increased at the subacute time point following concussion, and this elevation may persist chronically up to 1 year. Our study also suggests that concussion may be associated with a delayed decrease in Ins that is not seen in the acute or subacute time period. These results could inform future studies investigating potential therapeutic targets or prognostic biomarkers, which are critically needed for concussion generally, but also specifically in pediatric patients who may be particularly vulnerable to these neurological insults.

Our study adds to a small but growing body of literature using ^1^H MRS in pediatric concussion research. In 2012, Maugans et al. were the first group to report MRS findings in the adolescent population. Using single-voxel spectroscopy, they found no longitudinal changes in thalamus, frontal GM, or frontal WM NAA, NAA/Cr, or lactate in mild TBI, and no differences compared to controls (21). Their study looked at three time points, and final imaging for all subjects occurred < 3 months from the initial injury. While our findings align with their observations of NAA, we included imaging done approximately 1 year from injury, which offers further insight into the neurometabolic processes when symptoms have resolved for most patients.

Other pediatric studies have shown variable results. Our Ins findings are congruent with work by Poole et al. who followed football athletes longitudinally over the course of a season and found decreasing Cr and Ins in the dorsolateral prefrontal cortex (22). Although Poole et al. did not assess a control group, which limits comparison to the present study, we also demonstrated decreased Ins over time in our sample. Ins has been proposed to be a glial marker, among other functions, and decreased concentrations may represent glial dysfunction (15). We did not find significant differences in NAA/Cho nor the absolute concentrations of NAA, Cho, and Cr. Other studies have found increased GABA/Cr in the frontal lobe at approximately 1 month following concussion compared to controls (23), decreased Cho in the prefrontal region 3 months following concussion as compared to controls (24), decreased NAA/Cr and NAA/Cho in the corpus callosum and parietal WM at various time points ranging from 3 to 12 months post-concussion as compared to controls (25). It is important to note that the methodology and findings of these five studies are highly variable, and further studies are needed to continue to reproduce results and more deeply evaluate the neurometabolic consequences of pediatric concussion.

We also report a novel finding of increased thalamic Glx/Cho at the subacute and chronic time points. The spectra for glutamate and glutamine overlap highly, and concentrations are generally correlated, so their combined concentrations (i.e., Glx) are reported in this study. On further inspection of the quality of the spectral fitting for Glx spectra, the average Cramér-Rao-lower-bound value for all Glx peaks was approximately 16%, which indicates a fair degree of certainty in the Glx concentrations. Current pathophysiologic models suggest an acute and transient period of neuronal excitotoxicity and glutamate release at the beginning of the neurometabolic cascade of concussion (7). It is possible that our acute time point did not occur soon enough after injury to detect this cascade. Additionally, our findings indicate a delayed increase in glutamate concentrations, which suggests that altered neurotransmission may persist chronically following concussion. It should be noted that the differences in absolute Glx concentrations were not significant. In fact, further inspection of the data in Table 2 shows increasing absolute Glx values and decreasing absolute Cho values, suggesting that the trend observed in Glx/Cho could be a product of these two insignificant, opposing trends.

Our study has several limitations. First, this study was underpowered to detect small changes in metabolite concentrations, and it remains possible that additional meaningful neurometabolic changes occur during the longitudinal course of pediatric concussion. Due to the small sample size, we did not include covariates in our statistical model, which limits the generalizability of our findings and allows for the possibility of confounders that may have impacted these results. For instance, while there is evidence that brain metabolites are relatively stable above 4 years of age, there may still be age-related effects on metabolites concentrations, and this remains an important area for future research (30, 31). Given the limited sample size, we did not correct for multiple comparisons, and as a result our findings are preliminary and require confirmation with better-powered studies. Second, normal ranges for absolute concentrations are not well established in either pediatric or adult populations, making it difficult to interpret effect sizes. Though we believe our methodology for absolute concentration of metabolites was suitable for the longitudinal analysis we undertook, concentrations we report for GM would need to be refined for comparison to other studies that make different assumptions about metabolite T_2_ values or tissue water concentrations. Finally, obtaining estimates of the GABA and lactate concentrations will also be explored in future studies, as we expect that their values might change in the event of concussion. To obtain reliable estimates of these metabolites though, we need to employ special spectral editing MRSI acquisition techniques [e.g., MEGA-PRESS 2DJ (32) or 2D COSY MRSI (33)] in order to be able to uncouple the GABA and lactate signals from the other metabolites that are spectrally overlapping with them.

The current results align with the adult literature, which has shown minimal differences in ^1^H MRS measurements in the acute concussive period. Our study suggests that there may be significant longitudinal neurometabolic changes in the pediatric population leading to differences detectable beyond the acute time point, and we add to previous ^1^H MRS studies of pediatric concussion by examining the chronic time point at 1 year post-concussion. It remains to be seen how these results will fit with hypothesized biological pathways implicated in pediatric concussion, and larger sample sizes are needed to further evaluate smaller effects and to determine if the observed neurometabolic changes are reproducible.

## Data Availability

The datasets generated and analyzed for this study are available on request to the corresponding author.

## Ethics Statement

This study was carried out in accordance with the recommendations of the Institutional Review Board at Boston Children's Hospital with written informed consent obtained from all subjects. For minors, consent was obtained from parent proxies. All subjects or parent proxies gave written informed consent in accordance with the Declaration of Helsinki. The protocol was approved by the Institutional Review Board at Boston Children's Hospital.

## Author Contributions

PG, RM, and BG designed and supervised data collection. EM, JS, AC, and RM were responsible for imaging analysis design. EM, JS, and AC conducted the experiments. EM analyzed the results. RM and AC provided statistical analysis guidance. EM, JS, and AC wrote the manuscript, and all authors reviewed the manuscript.

### Conflict of Interest Statement

The authors declare that the research was conducted in the absence of any commercial or financial relationships that could be construed as a potential conflict of interest.
